# 基于无基底扣除的数据趋势累积谱峰检测算法

**DOI:** 10.3724/SP.J.1123.2020.11009

**Published:** 2021-06-08

**Authors:** Menghan JIA, Zhaoyan HUI, Hui ZHANG, Yu GAO, Meiqi TONG, Yinan MA

**Affiliations:** 北京科技大学能源与环境工程学院, 北京 100083; School of Energy and Environmental Engineering, University of Science and Technology Beijing, Beijing 100083, China; 北京科技大学能源与环境工程学院, 北京 100083; School of Energy and Environmental Engineering, University of Science and Technology Beijing, Beijing 100083, China; 北京科技大学能源与环境工程学院, 北京 100083; School of Energy and Environmental Engineering, University of Science and Technology Beijing, Beijing 100083, China; 北京科技大学能源与环境工程学院, 北京 100083; School of Energy and Environmental Engineering, University of Science and Technology Beijing, Beijing 100083, China; 北京科技大学能源与环境工程学院, 北京 100083; School of Energy and Environmental Engineering, University of Science and Technology Beijing, Beijing 100083, China; 北京科技大学能源与环境工程学院, 北京 100083; School of Energy and Environmental Engineering, University of Science and Technology Beijing, Beijing 100083, China

**Keywords:** 无基底扣除, 离散差分, 趋势累积, 遍历寻峰, 谱峰检测算法, no base deduction, discrete difference, trend accumulation, searching for peaks by traversing, peak detection algorithm

## Abstract

谱峰的检测分析在色谱技术研究中具有十分重要的作用,但在色谱数据采集、传输的过程中,不同程度的噪声干扰给谱峰检测带来了极大的困难。目前传统的谱峰检测算法普遍通过基底扣除的方式对谱峰的形态进行预定义,将谱峰分为单峰、重叠峰等多个种类。针对不同种类的谱峰采用不同的检测方法,这就导致了传统的谱峰检测算法具有高复杂度、低自动化程度以及容易失真等缺点。因此,该文从另一个角度出发提出了一种新型的谱峰检测算法。该算法取消基底扣除以及谱峰分类这一步骤,直接在源数据曲线的基础上进行谱峰检测,主要分为离散差分、趋势累积以及遍历寻峰3个步骤。首先通过信号量表征数据升降趋势;然后进行数据趋势累积,根据累积总和定位谱峰,采用三点定位的方式,即峰起点、极值点和峰终点描述一个谱峰的位置;最后根据遍历排序的方式进行谱峰的筛选。此外,通过谱峰扣除的方式得到曲线基底部分。采用C语言设计编写了算法程序,并对多个动态比表面积分析仪测定的色谱图进行了检测分析,结果显示使用该算法可以精准区分谱峰部分与基底部分,受数据曲线毛刺、震荡等噪声干扰很小,谱峰的三点定位十分准确,且不受其复杂形态的影响,具有很强的普适性。与其他算法相比,该算法定位准确,结构清晰,具有较好的稳定性以及可靠性。该文报道了无基底扣除以及趋势累积等新型谱峰检测思想在吸脱附色谱曲线中的应用,证明了其在吸脱附色谱峰检测中的有效性和良好的应用前景。

## Abstract (en)

The detection and analysis of spectral peaks play an important role in research on chromatography technology. However, in the process of collecting and transmitting chromatographic data, it is very difficult to detect spectral peaks owing to the interference of different levels of noise. Most of the traditional spectral peak detection algorithms follow three steps: spectral smoothing, baseline correction, and spectral peak recognition, which require high denoising and curve smoothing, and therefore increase the complexity of the algorithm. In addition, a traditional spectrum peak detection algorithm generally defines the shape of the spectrum peak by applying the base deduction method, and divides the spectrum peak into a single peak, overlapping peaks, and so on. Different detection methods are used for different types of spectral peaks, which lead to the shortcomings of traditional peak detection algorithms, such as high complexity, low automation, and susceptibility to distortion. Therefore, this study proposes a novel peak detection algorithm developed using a different point of view. The algorithm omits the base subtraction and spectral peak classification steps and instead detects spectral peaks directly based on the source data curve. In a traditional spectrum peak detection algorithm, the spectrum peak classification depends on determining a baseline. If the baseline is adjusted, the baseline will fit the spectrum peak more closely. At this time, the overlapping peaks can be regarded as two connected peaks. However, there is no so-called baseline in the source data curve, and therefore the proposed algorithm cannot classify the spectral peaks using the baseline approach. Instead, an obvious bulge or depression in the source curve is considered to be the spectral peak. This algorithm essentially performs three steps: discrete difference, trend accumulation, and searching for all peaks. First, the difference between adjacent data is obtained using a discrete difference process. The difference value is compared with 0, and either a 1 or -1 value is used to replace the difference value to reflect the data fluctuation trend. The signals representing the trend are accumulated, and the spectrum peak is located according to the sum of the accumulated signals. The algorithm uses three-point location; that is, the peak starting point, extreme point, and peak end point are used to describe the position of a spectral peak. Finally, according to the spectrum peaks obtained in the previous step, the magnitude of each peak is calculated, and the spectrum peaks are screened by a sorting method. In this manner, the algorithm skips the base subtraction part and obtains the spectrum peak directly. Therefore, to obtain the base part, the peak subtraction method is applied. This study used the C language to design and write the algorithm, and nitrogen adsorption and desorption chromatographic curves measured by several dynamic specific surface area analyzers were detected and analyzed. The results indicate that the proposed algorithm can accurately distinguish the peak part from the base part, and is robust to data curve burr, vibration, and other types of noise. The three-point location of the spectrum peak is very accurate and is not affected by its complex morphology. Therefore, it has strong universality. Compared with other algorithms, this algorithm has the advantages of accurate positioning, clear structure, and good stability and reliability. The application of the proposed peak detection methods such as base-free deduction and trend accumulation, in the adsorption and desorption chromatographic curve and has been proven effective in the determination of absorption and desorption chromatographic peaks.

随着色谱技术的发展,谱图的分析检测技术也日益提高^[1]^。人们提出了许多谱峰检测的方法,常见的有幅值法^[2]^、一阶导数法^[3,4]^、二阶导数法^[5]^、小波变换法^[6,7,8]^、曲线拟合法^[9,10,11]^和遗传算法^[12]^等。幅值法实现简单,但过度依赖于阈值的确定,准确率很低,较少被采用;一阶、二阶导数法需要数据光滑连续,受波动的影响大,不适合离散采集数据;小波变换法和曲线拟合法均需要人工干预,且需要操作者具备良好的专业知识储备以及经验积累,给实际应用带来一定的困难;遗传算法对于经验公式有很强的依赖,误差很大。

目前已有的谱峰检测算法大部分围绕谱线平滑、基线校正与谱峰识别3个步骤^[13]^,对数据去噪、曲线平滑的要求很高,增大了算法的复杂度以及难度。本文提出一种跳过谱线平滑与基线校正两个步骤,在无基底扣除的情况下,利用数据趋势累积的原理进行谱峰定位的算法,简称趋势累积谱峰检测算法。该算法稳定可靠,准确度高且普适性强,具有较为广泛的应用前景。

## 1 算法原理

谱图不同位置的峰代表着不同的信息。对于色谱图,特定位置的峰代表某一组分,物质含量与峰面积相关;对于动态比表面仪,特定位置的峰代表着气体吸附或者脱附,气体吸脱附量与峰面积相关。因此,确定峰起点、极值点和峰终点对于谱图的分析十分重要。趋势累积谱峰检测算法计算过程如图1所示,首先通过差分过程获得数据的升降趋势,再根据趋势的累积定位谱峰起点、极值点和峰终点,计算所有谱峰的峰值,通过排序的方法对谱峰进行筛选,最终获得精准的谱峰信息。趋势累积谱峰检测算法的验证实验均采用动态比表面分析仪在不同条件下测得的吸脱附色谱图。文中所有曲线图的数据均取自于空样品管吸脱附氮气色谱曲线,实验中铜管采用70~65 ℃水浴,热导池为恒温50 ℃。

**图 1:**
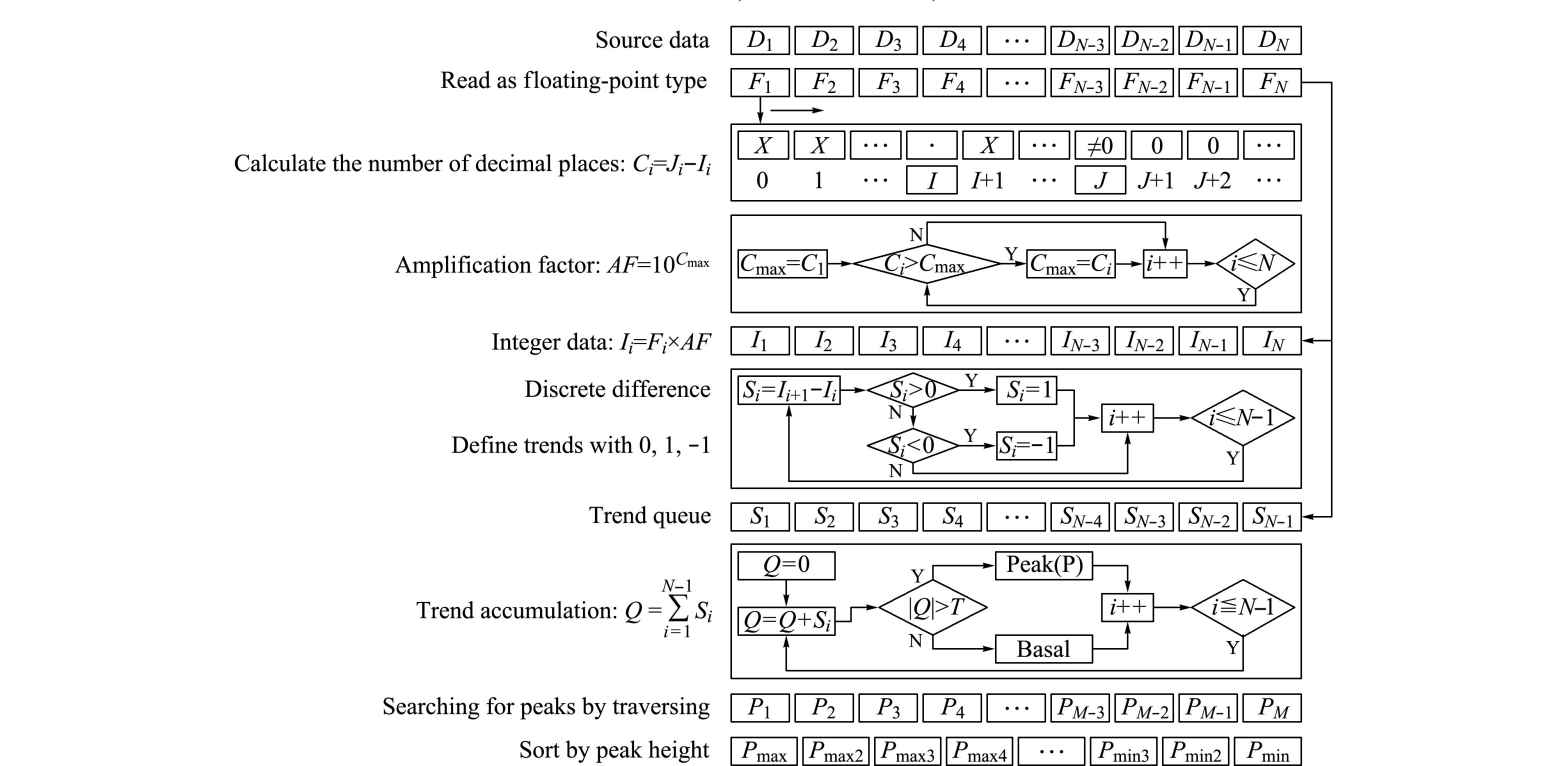
趋势累积谱峰检测算法计算流程图

### 1.1 数据预处理

浮点型数据存储空间大,运算时间长,不利于嵌入式终端的快速数据处理。为了减少运算量,对数据进行浮点型转整型的预处理操作,如图1所示,由于事先不知采样数据类型,为了防止漏读、错读,前期均以浮点型格式读入采样数据队列:*F*_1_, *F*_2_, *F*_3_, …, *F_i_*(*i*=1, 2, 3, …, *N*; *N*为采样点数),得到采样队列范围(*F*_min_, *F*_max_)。

浮点型转整型预处理不可直接进行强制转换,虽然在采样队列为整数的情况下不会产生较大的误差,但对于小数采样队列,其小数位在转换过程中将被全部舍去,数据可能会出现严重的偏差。例如:采样数据均是小于0.1的数,即0.01或者0.09,这种情况下如果进行强制转换,则采样数据全部为0,数据失去本来特征,后续运算将无法进行。因此应先将源数据乘以放大因子进行放大,以确保后续转换不会影响数据的精度和运算。根据采样队列所有数据中最后一位非0小数位与小数点的距离的最大值*C*_max_即可确定放大因子。在不影响计算精度的情况下可适当对放大因子进行调整。

### 1.2 数据去噪

采样数据在产生、激励或传输的过程中,会受到不同程度的噪声干扰,特别是当采样信号差值较小时,噪声干扰的情况显得尤为严重,直接影响采样信号特性的准确识别,出现曲线毛刺过多、基线不稳定、拖尾峰等现象,对峰识别算法造成干扰,导致漏判或误判。因此,减少噪声对谱峰检测算法的干扰至关重要,数据去噪要求能够去除局部明显的噪声峰和曲线上的毛刺,平滑曲线,改善信噪比,增加谱峰检测的准确性。

目前已有很多数据去噪的算法,例如限幅滤波、中值滤波、递推中值滤波、均值滤波等^[14]^。趋势累积谱峰检测算法对数据去噪的要求是滤除明显的毛刺,无需进行光滑处理,故采用递推中值滤波法^[15,16]^,主要原理是把连续*N*个采样值看成一个队列,队列的长度固定为*N*,将队列中的*N*个数据进行排序,取队列中间位置的数值作为有效值输出,再将采样得到的新数据放在队尾,替换掉原队列首部的数据,遵循先进先出原则,保持队列长度始终为*N*。该滤波算法优点是曲线不会失真,适用范围广泛;但由于排序次数多,运算速度较慢。

### 1.3 离散差分

事实上,计算机并不像人一样主观判断峰的形状与位置,只能在大量的数据运算中寻找符合特征描述的数据,这就是谱峰检测算法。谱峰检测在大量的数据分析中十分重要,研究人员基于不同峰的特征对峰进行了分类和描述,定义了峰曲线上的一些特殊点作为特征点,峰的特征点包括峰的起点、前拐点、极值点、后拐点及终点,谱峰检测就是对这些特征点的识别。

理想情况下,谱图曲线的基底部分是一条水平直线,而谱峰部分则是先逐渐上升(下降)后逐渐下降(上升)的曲线,与基底部分相比,谱峰部分存在数据升降的趋势,根据这种升降趋势对基底与谱峰进行区分。

离散差分过程是获得数据变化趋势的过程,对预处理后的数据队列*I*_1_, *I*_2_, *I*_3_, …, *I_i_*(*i*=1, 2, 3, …, *N*; *N*为采样点数)进行差分,*S_i_*=*I_i_*_+1_-*I_i_*, *S_i_*为前后两个数据的差值。

若*S_i_*大于0,表示数据增大,则令*S*_i_为1,表示数据上升趋势;若*S_i_*小于0,表示数据减小,则令*S_i_*为-1,表示数据下降趋势;若*S_i_*等于0,表示数据不变,用0来表示平稳趋势;如图2b所示。通过离散差分过程可得到数据趋势队列:*S*_1_, *S*_2_, *S*_3_, …, *S_i_*。

**图 2:**
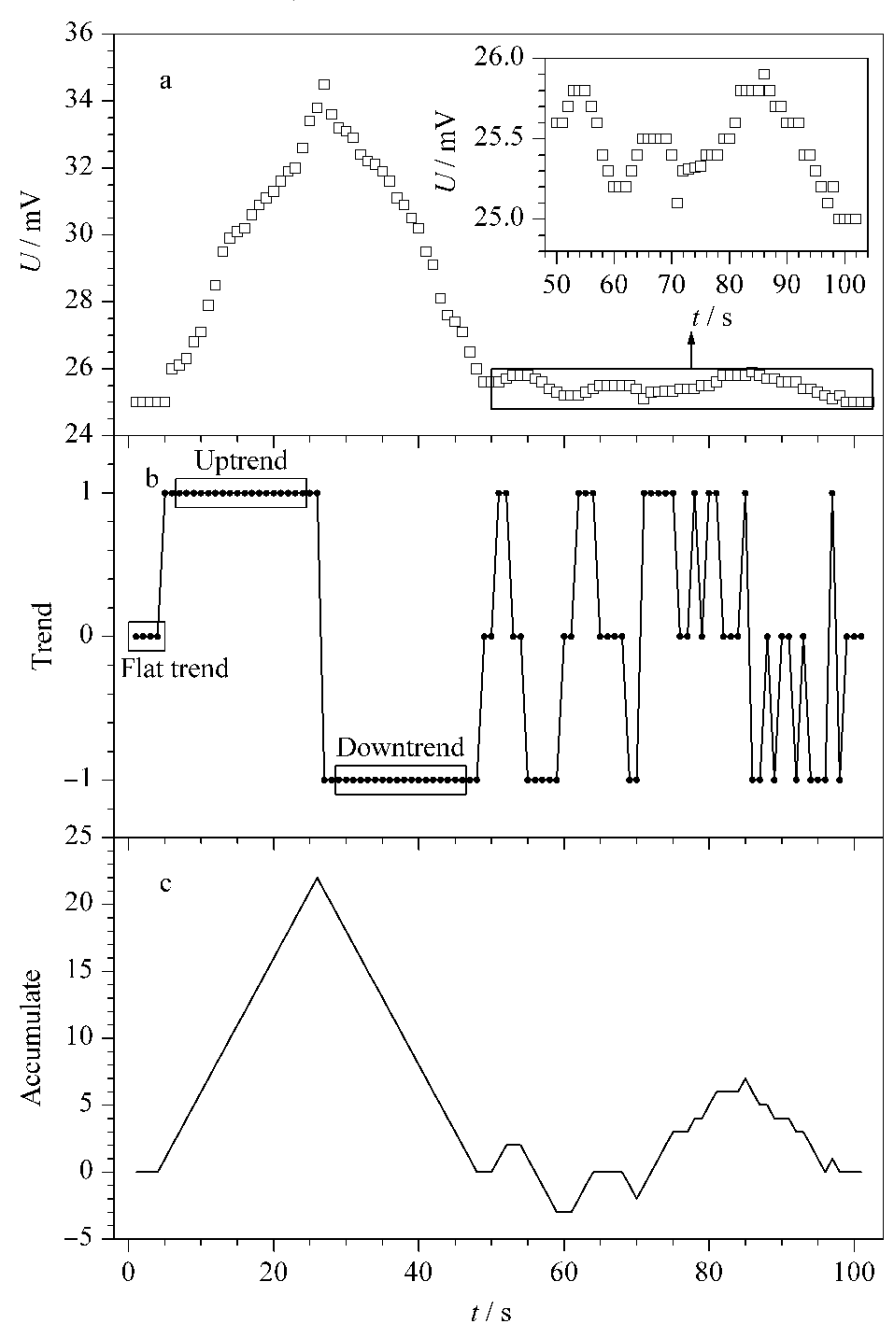
尖峰段、基底段源数据队列与差分、累积队列对比图

将数据队列和趋势队列进行对比,如图2中a、b和图3中a、b所示。根据图2b可以看出,曲线基底部分经过离散差分后得到的趋势队列处于震荡状态。尖峰部分经过离散差分后得到的趋势队列规律十分明显,其前半部分持续上升,后半部分持续下降。这类峰形是比较理想化的,实际上大多数峰都像图3b中的缓峰一样,震荡上升或下降,或者在持续上升、下降的过程中夹杂出现部分下降、上升趋势。这样的峰形仅从趋势队列来看与图2b中基底部分十分相似,难以区分,这两种情况的出现使得谱峰检测过程十分困难。

**图 3:**
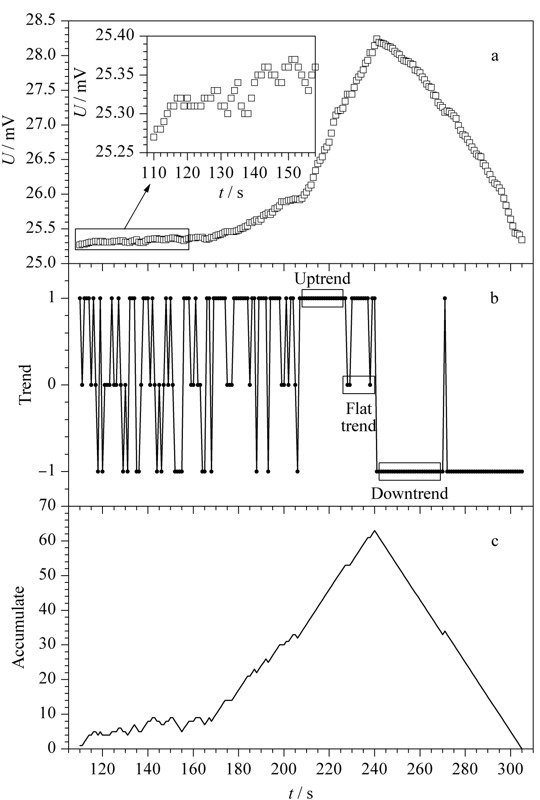
缓峰源数据队列与差分、累积队列对比图

### 1.4 趋势累积

在理想情况下,通常根据升降趋势的产生、结束确定谱峰的位置,但实际的谱图中绝大多数情况下都会出现基底震荡以及缓峰的现象。针对这种情况,仍然采用升降趋势对谱峰进行定位,通过趋势累积判断趋势的产生和结束。

如图2b所示,数据曲线上谱峰部分的趋势队列会先出现大量的上升趋势,后出现大量的下降趋势,形成一个完整的谱峰;而数据曲线上的基底部分,由于存在一些噪声干扰的缘故,不会呈现完全持平的趋势,但绝大多数的基底部分均是在极少量的上升或者下降趋势之间往复摆动,随着时间的延长,两者相互抵消,形成稳定的平稳趋势。趋势累积根据数据谱峰段与基底段趋势上的数量差异对谱峰进行识别,趋势的数量Sum则是通过数据趋势队列*S*_1_, *S*_2_, *S*_3_, …, *S_i_*(*i*=1, 2, 3, …, *N*-1; *N*为采样点数)的累加获得。

根据趋势的数量Sum对谱峰检测的原理是:在理想情况下,当Sum大于0的时候,判断为峰起点;当Sum达到最大值的时候,判断为峰值点;当Sum重新归零的时候,判断为峰终点。如图2c所示,对于理想的尖峰,这种判断模式可以准确地检测谱峰。除此之外,该模式还可准确检测到非理想的缓峰,如图3c所示,其优点在于对谱峰的检测不会遗漏,适用范围广,这也是该谱峰检测算法较其他算法更为普适的原因之一。对于图2尖峰后面的基底部分,按照这种判断模式也会检测出谱峰,说明这种模式的缺点在于区分基底与谱峰的效率低。根据图2c可看出基底段趋势的数量Sum最大值较谱峰段小很多,针对Sum最大值设置阈值的方法可以区分基底和谱峰。

### 1.5 遍历寻峰

趋势累积的方法可以检测出所有的峰,但并不能完全区分基底段与谱峰段,原因在于阈值的设定并不能做到准确无误。如图4所示,AI、BI、CI 3种情况均被认为是峰,但在源数据上A与B和C有较大的差异,从源数据上可以看出A为基底,B和C是谱峰,这种情况下,阈值的设定无法准确无误的将A区分出来。为了精准识别,采用遍历寻峰的方法对谱峰进行筛选。首先计算检测到的所有谱峰峰值点与峰起点的差值,然后进行排序,众数小且差值大的为所要检测的谱峰。

**图 4:**
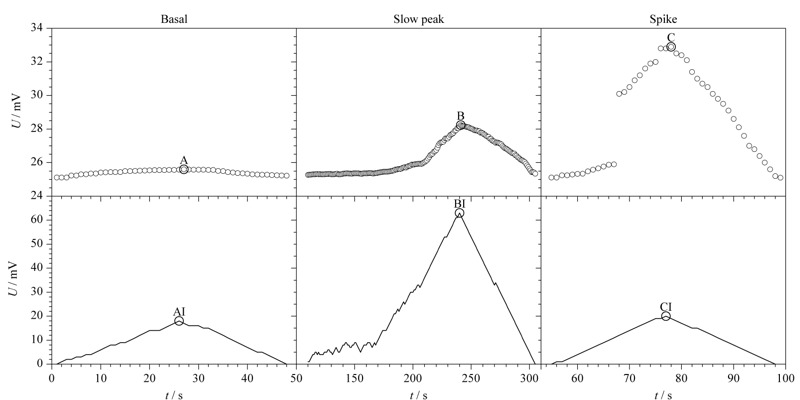
谱峰段趋势累积效果图

如果要遍历所有谱峰,将其进行一遍排序,会增加计算机的工作量,降低算法的运算速度。为提高运算速度,降低工作量,可添加人工干预,人工确定谱峰的数量,根据得到的谱峰数量从大到小进行取舍,无需大量排序统计。

## 2 算法核心程序设计

算法程序采用C语言编写,核心在于离散差分过程以及趋势累积过程,遍历寻峰是一个排序统计的过程,程序复杂度较低。

图5为离散差分程序流程图,图中右侧为程序的N-S图,采用顺序、选择和循环结构展示程序运行逻辑;左侧为对应的程序核心源代码,实现数据的放大、差分、覆盖等功能。可加入智能化的用户模块,根据用户的需求调整数据放大因子。

**图 5:**
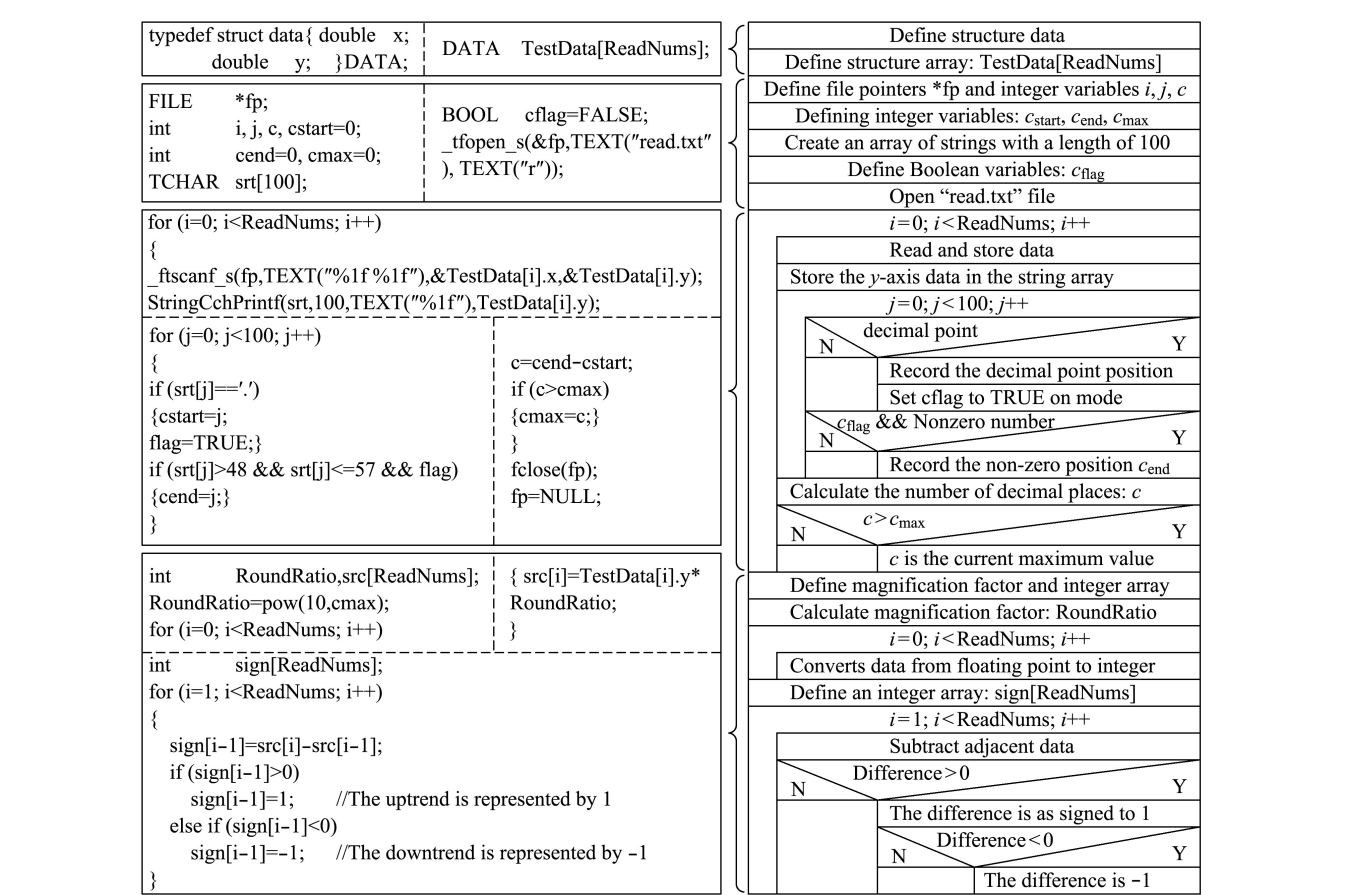
离散差分程序流程图

图6为算法趋势累积程序流程图,同样采用源程序与N-S图相结合的模式。由于本程序谱峰数量未知,故采用链表结构进行谱峰数据的记录,链表结构可以克服数组需要预先知道数据大小的缺点,充分利用计算机内存空间,实现灵活的内存动态管理。本程序在链表中记录谱峰各个点在源数据数组中的编号,后期使用简单方便且节省空间。图6算法程序中将趋势累积过程阈值设置为3,实现基底与谱峰的第一步区分。关于这里的阈值,可添加用户智能设置模块,用户可以通过对比观察对阈值进行修改。

**图 6:**
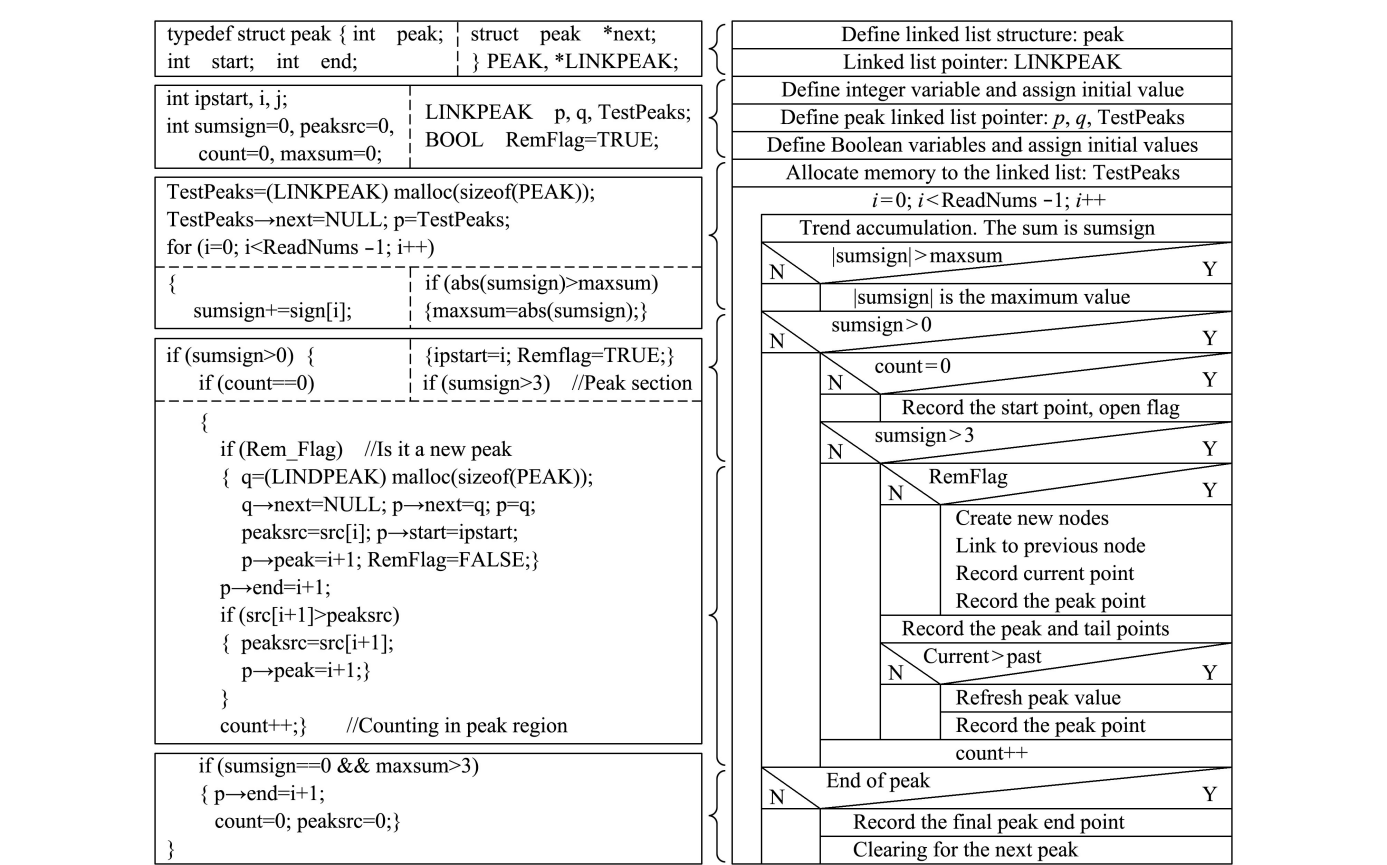
趋势累积程序流程图

## 3 分析与讨论

为验证算法程序的可行性,选择动态比表面积分析仪测定的吸脱附谱图进行测试。图7为运行算法程序得出的谱峰识别效果图。通过程序去除识别出来的谱峰部分,得到谱图基底部分效果图,见图8。

**图 7:**
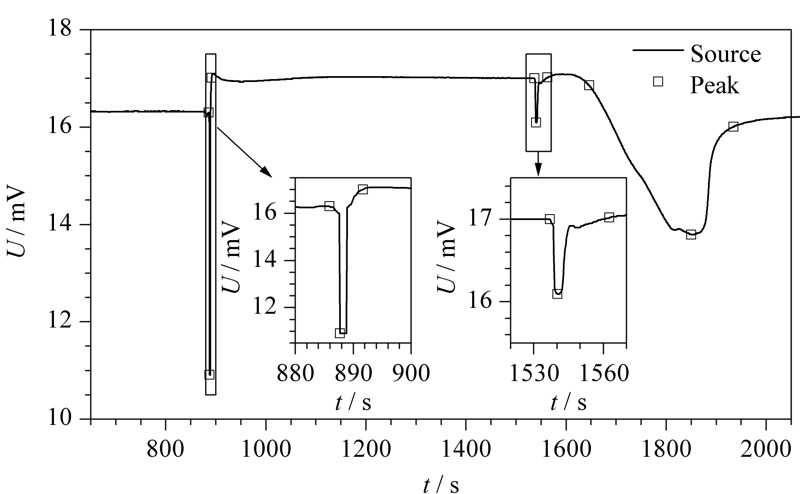
趋势累积谱峰检测算法程序的谱峰识别效果图

**图 8:**
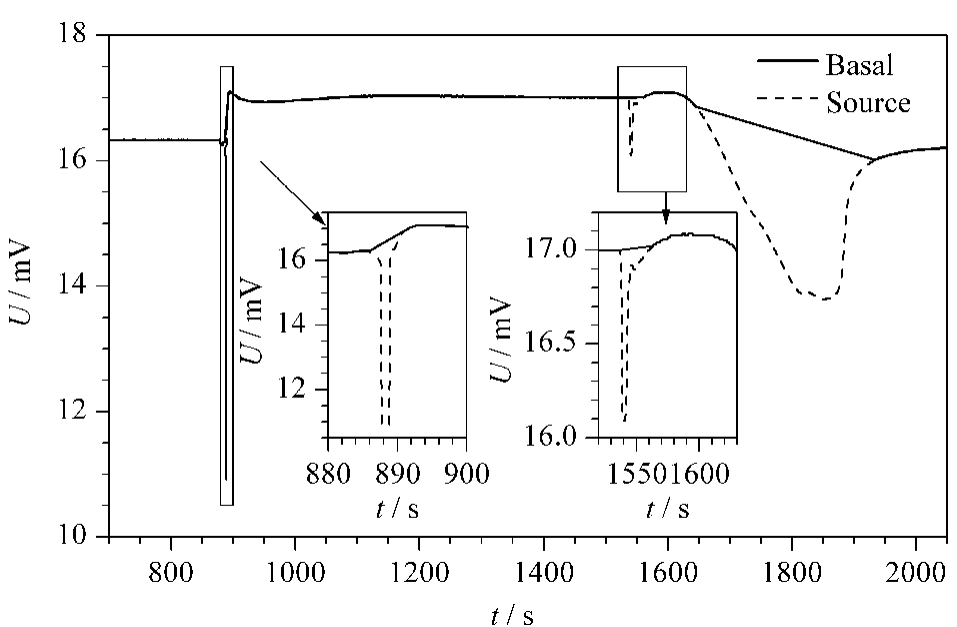
趋势累积谱峰检测算法程序的基底部分效果图

从图7可以清晰地看出,本文的趋势累积谱峰检测算法程序检测得到的谱峰起点、峰值点以及终点的位置十分准确,且不受数据曲线毛刺、震荡的影响。对于图7中第1个尖峰尾部基线上升的情况也没有出现峰终点定位不准的现象,这表明该算法并不受基底的影响。

趋势累积谱峰检测算法中检测的对象是谱峰,也就是连续的上升(下降)趋势和连续的下降(上升)趋势。传统的谱峰检测算法是根据基底的扣除将谱峰进行分类归纳,分为单峰、拖尾峰、重叠峰等^[17]^,这实际上为谱峰检测增添了困难。本算法的不同之处就在于不经过基底扣除这一步骤,直接对谱峰进行检测,所有的谱峰只分为急剧变化的尖峰和震荡变化的缓峰两大类,大大降低了算法的复杂度,同时也保证了定位的准确性,在尖峰、缓峰的识别上都取得了良好的效果。只要定位到各个谱峰的位置,扣除谱峰部分就可以得到基底部分,根据图8可以看出通过谱峰扣除得到的基底部分也十分贴合原曲线。

为验证趋势累积谱峰检测算法程序的准确性以及可靠性,选择其他两种已有的谱峰检测算法与本算法进行比较,依旧选取动态比表面积分析仪测定的吸脱附谱图进行测试。如图9所示,一阶导数法除了变化迅速且急剧的尖峰可以准确识别之外,其他类型的谱峰均无法识别。二阶导数法对于尖峰的识别较为准确,但对于缓峰则无法识别。综合来看,趋势累积谱峰检测算法识别效果最为准确且较其他二者更为普适。

**图 9:**
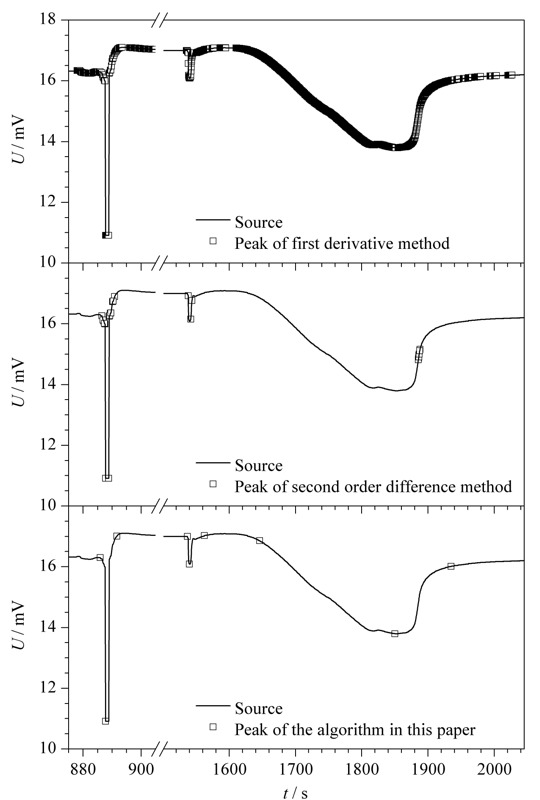
趋势累积谱峰检测算法与其他算法识别效果对比图

## 4 结论

本文提出了一种在无基底扣除的情况下基于数据趋势累积原理的新型谱峰检测算法,通过算法程序的设计编写以及大量数据的验证改进,总结得到以下结论:用整型信号量表征数据升降趋势,采用信号量进行后续计算,对源数据的预处理过程要求较低,并且降低了算法的复杂度;将离散差分得到的趋势数据累积,巧妙地实现了谱峰的精准定位,计算过程简便且快速,针对复杂度很高的缓峰也能取得较好的效果,算法的普适性很高;将设置阈值与谱峰排序相结合,实现了谱峰与基底的分离,分离效果较好,算法稳定且准确度高。该算法适用于大部分谱图的谱峰检测分析,但在峰终点的定位上还需采用大量不同类型的数据进行验证分析,根据实验结果改进算法程序。
